# Joint position sense in individuals with anterior knee pain

**DOI:** 10.4102/sajp.v77i1.1497

**Published:** 2021-02-18

**Authors:** Carlyn Rhode, Quinette A. Louw, Dominique C. Leibbrandt, Leone Williams

**Affiliations:** 1 Division of Physiotherapy, Department of Health and Rehabilitation Sciences, Stellenbosch University, Cape Town, South Africa

**Keywords:** patellofemoral pain, proprioception, movement analysis, risk factors, assessment, rehabilitation

## Abstract

**Background:**

Anterior knee pain (AKP) commonly affects both physically active and sedentary individuals and the aetiology is unknown. Altered joint position sense (JPS) impacts accurate motor action and knee joint stability. It is unclear whether people with AKP have altered JPS.

**Objective:**

The aim of our study was to investigate JPS in the knees of individuals with AKP.

**Method:**

A descriptive cross-sectional study measured JPS in 25 participants with unilateral or bilateral AKP. JPS was measured using active JPS testing during single leg squat (SLS) and active knee extension (AKE) in sitting. Target angles (TA) were self-determined based on each participant’s capabilities. The absolute error (AE) was the main outcome measure. Impaired JPS was classified as an AE equal to or greater than five degrees.

**Results:**

There were no significant differences in JPS when comparing the affected and unaffected knees in participants with AKP (*p* > 0.05). However, a subgroup of participants with altered knee JPS was identified. There was a tendency towards greater knee flexion in the TAs of knees without AKP.

**Conclusion:**

Our results showed that JPS is not significantly more impaired in knees with AKP compared with knees without AKP in a group of individuals with AKP. A subgroup with altered JPS in knees with and without AKP was identified. This finding could be because of compensatory gait patterns and the precision of the Vicon 3D motion analysis system.

**Clinical implications:**

Joint position sense should be assessed bilaterally in individuals with AKP.

## Introduction

Anterior knee pain (AKP) is a common condition affecting the knee joints of both young, physically active and sedentary individuals (Crossley et al. 2016). Anterior knee pain accounts for 25% – 40% of all knee problems presenting at sports medicine clinics; one in four of an active population is affected, leading to chronic knee pain among young adults (Coppack, Etherington & Wills 2011; Crossley et al. 2016; Dutton, Khadavi & Fredericson 2016). Anterior knee pain has a higher prevalence amongst women, with an incidence two to three times more than that of men (Dutton et al. 2016; Prins & Van Der Wurff 2009).

Anterior knee pain is characterised by anterior, peri-patellar or retro-patellar pain with an insidious onset and is exacerbated under conditions of increased patellofemoral joint (PFJ) stress (Dutton et al. 2016; Nunes et al. 2013). Aggravating activities include climbing or descending from stairs, prolonged sitting, squatting and running (Nunes et al. 2013). The aetiology of AKP is unclear with a possible multi-factorial nature and develops secondary to functional or structural mal-alignment of the PFJ (Green et al. 2014). Management of AKP remains challenging with 91% of patients with AKP reporting persistent symptoms after extended follow-up and medical management (Dutton et al. 2016). It is, therefore, important to understand the aetiological pathways that may cause the pain.

Altered proprioception has been documented in patients with AKP (Akseki et al. 2008; Baker et al. 2002; Cyrillo & Cabral 2014; Guney et al. 2015). Proprioception is ‘the use of joint position sense (JPS) and joint motion sense to respond to stresses placed upon the body by alteration of posture and movement’ (Norris 2011).

Clinical symptoms of altered proprioception may include disturbed balance and clumsiness because of disturbed motor function and joint reflex stabilisation. It has been proposed that the altered proprioception amongst individuals with AKP can result in recurrent and persistent pain, and that this could increase the risk of joint osteoarthritis in the long term (Clark, Röijezon & Treleaven 2015; Röijezon, Clark & Treleaven 2015). Altered proprioception in individuals with AKP could be as a result of mechano-receptor damage associated with abnormal movement patterns and motor reactions (Clark et al. 2015; Han et al. 2016; Hillier, Immink & Thewlis 2015).

The altered movement patterns and motor reactions may be underpinned by weak quadriceps muscles, altered timing of the vastus medialis oblique muscle and altered tissue flexibility of the quadriceps and hamstring muscles (Kaya et al. 2010; Lankhorst, Bierma-Zeinstra & Van Middelkoop 2012). Altered motor reaction in AKP may be because of altered proprioceptive input from muscle spindles in skeletal muscles (Röijezon et al. 2015). Muscle spindles in skeletal muscles are a type of mechano-receptor and a major source of proprioceptive feedback (Röijezon et al. 2015). Another possible reason for altered proprioception in a population with AKP could be small nerve damage in the lateral retinaculum of the patella (Sanchis-Alfonso & Rosello-Sastre 2003). This nerve damage in the lateral retinaculum may be because of the mal-tracking of the PFJ (Sanchis-Alfonso & Rosello-Sastre 2003). It is unclear whether altered proprioception could be a risk factor leading to the onset of AKP or a resulting factor contributing to the chronicity of the condition.

The ability to accurately sense joint position is essential to aid an individual’s response when the body encounters stress (Smith, Davies & Hing 2013). Joint position sense testing can be conducted under active (biasing joint mechanoreceptors) or passive (stimulating joint and muscle tendon mechanoreceptors) conditions (Roijezon et al. 2015). Joint position sense is the ability of an individual to accurately reproduce a target angle (TA) (Baker et al. 2002; Selfe et al. 2006). The most common clinical metric used to assess JPS is absolute error (AE). Absolute error refers to the difference between the TAs and the response angle (Han et al. 2016; Hillier et al. 2015; Ogard 2011; Röijezon et al. 2015).

There is limited research investigating JPS in individuals with AKP (Yosmaoglu et al. 2013). Previous studies in this field reported no significant differences in JPS between individuals with and without AKP (Bennell et al. 2005; Naseri & Pourkazemi 2012; Yosmaoglu et al. 2013). In contrast, other studies have observed that JPS is significantly impaired in individuals with AKP compared with controls (Akseki et al. 2008; Baker et al. 2002; Cyrillo & Cabral 2014; Guney et al. 2015). The reasons for these conflicting findings are unknown and although all these studies measured JPS, different methods for measuring JPS were used. These methods included: (1) image recorded where photography is used to assess knee joint angles (Baker et al. 2002; Naseri & Pourkazemi 2012), (2) electro-goniometry with knee angular error (Akseki et al. 2008; Cyrillo & Cabral 2014), (3) dynamometry (Guney et al. 2015) and (4) a functional loaded squat system (Yosmaoglu et al. 2013).

Three studies measured non-weightbearing (NWB) JPS only (Akseki et al. 2008; Guney et al. 2015; Yosmaoglu et al. 2013), whereas the other studies combine weightbearing (WB) and NWB. Weightbearing positions have been shown to be more accurate; however, NWB knee JPS has the greatest potential for isolating the proprioceptive status of the knee joint only (Stillman & McMeeken 2001). There were also differences in the included study samples. The studies that found no significant differences used very specific populations that included pain-free participants with induced AKP (Bennell et al. 2005), athletes (Naseri & Pourkazemi 2012) and women (Yosmaoglu et al. 2013). In the studies with significant differences, activity levels were not monitored.

However, in these studies (Akseki et al. 2008; Baker et al. 2002; Cyrillo & Cabral 2014; Guney et al. 2015) participants presented with a mean duration of symptoms ranging from 3 to 36 months, indicating sub-acute ongoing chronic pain. Two of the studies focused on females only (Cyrillo & Cabral 2014; Guney et al. 2015) and one study (Guney et al. 2015) required participants to present with at least moderate pain levels (more than 4/10) that were present daily for at least a month.

The variation in methodology could also be because of the lack of a gold standard test to assess JPS. Furthermore, the tests used in these studies have poor clinical applicability and are poorly evaluated and reported on by the respective authors (Guney et al. 2015). Current studies use 1D and 2D systems only. It is possible that these methods do not have adequate sensitivity to detect small changes in the AE. The VICON 3D movement analysis system is considered the gold standard objective measure for 3D postural analysis (Brink et al. 2013).

An additional problem with the current evidence is the lack of a clear classification system of what constitutes ‘good’ or ‘poor’ JPS. The current evidence on participants with AKP only compares differences between AKP and control groups with no clear interpretation of what the values mean. Relph and Herrington (2016) assessed 116 healthy, pain-free participants between the ages of 18 and 82 years. They assessed the normative JPS AE values into flexion and extension. The mean AE values ranged between 3.1 and 3.9 degrees for flexion and 2.5 and 3.9 degrees for extension. The minimal detectable difference for JPS has been reported to range from 1.23 to 2.14 degrees in AE scores (Relph & Herrington 2015). Clark et al. (2016) reported that AE scores range between 3.18 and 5.97 degrees in healthy pain-free adults. In healthy participants, Callaghan et al. (2002) regarded an error of less than five degrees as good proprioception and an error of more than five degrees as poor proprioception.

It is unknown how much of a difference in AE is required to result in an increased injury risk. However, based on the recommendations from these studies (Callaghan et al. 2002; Clark et al. 2016; Relph & Herrington 2015) we used an AE greater than five degrees as an indication of poor JPS for the purpose of our study.

More research is needed to establish if individuals with AKP present with altered knee JPS as this could potentially lead to altered movement patterns and potentially chronic symptoms. Clinicians need to know whether to address JPS in rehabilitation when treating patients with AKP. The aim of our study, therefore, was to determine if JPS is altered in the knees of individuals with AKP. Our study is the first to use the Vicon 3D motion analysis system to accurately measure knee JPS during WB in an AKP population.

## Methodology

A cross-sectional, descriptive study was used to collect data. All included participants presented with AKP, with either unilateral or bilateral AKP. Two groups were formed and the knees with and without AKP were compared. We conducted our study at the Human Movement Analysis Unit, Faculty of Medicine and Health Sciences, Stellenbosch University.

Sample recruitment was aimed to attract individuals with AKP from different socio-economic backgrounds, sporting codes and areas. Letters of invitation were sent to various universities, sports clinics, physiotherapy practices and sporting clubs. The advertisements requested for volunteers who were between the ages of 14 and 40, who had pain at the front of the knee during common aggravating activities such as squatting and stair climbing and no previous lower limb surgery. Potential participants emailed the research team and completed the screening questionnaire. Participants who complied with the AKP screening tool and the diagnostic checklist (Leibbrandt & Louw 2017), which was based on a systematic review of diagnostic studies and developed specifically for our study, were considered for inclusion.

Screening was undertaken by the first author (CR) who had been trained to use the screening tool by the author with experience in treating AKP (DL). Participants with unilateral and bilateral AKP were considered for inclusion. In cases where both knees were affected with AKP, both knees were tested for altered JPS and compared with knees without AKP.

### Sample size

We aimed to include 25 participants with AKP. This sample size was calculated using a pragmatic approach and based on previous studies (Akseki et al. 2008; Baker et al. 2002; Cyrillo & Cabral 2014; Guney et al. 2015).

### Measurement tools

#### Vicon 3D motion analysis system

The eight-camera Vicon T-20-series motion analysis system (Vicon Motion Systems Ltd., Oxford, UK) with Nexus 1.7 software was used to assess JPS. The Vicon has demonstrated high accuracy and reliability and has been shown to have less than a 1.5-degree error (Ehara et al. 1997; Richards 1999).

We used retro-reflective markers with a diameter of 9.5 mm. Dynamic calibration was performed according to standard laboratory protocol, and the Vicon T-wand was placed on a 3D Bertec force plate (Bertec Corporation Ltd.), which is synchronised with the Vicon motion analysis system.

#### H-Frame

An H-frame was constructed based on a study by Clark et al. (2016). The function of the H-frame was that of a range of motion (ROM) guide when establishing the TA for participants during the test trial.

The H-frame was positioned so that the rubber band (cross bar) made contact with the distal part of the patella during a single leg squat (SLS) and the crossbar touched the skin overlying the anterior ankle joint line during active knee extension (AKE). The H-frame was removed during the test procedure.

#### Anterior knee pain scale questionnaire

The anterior knee pain scale (AKPS) is a 13-item questionnaire used to determine functional ability in individuals with AKP. This scale is scored out of 100, with a higher score indicating less disability. The AKPS demonstrated high reliability and responsiveness in a population of patients with AKP (Crossley et al. 2004; Watson et al. 2005).

#### Numeric rating scale

This numeric rating scale (NRS) scale is a well-known outcome measure to evaluate levels or intensity of pain (Crossley et al. 2016). The NRS is scored from 0 (no pain) to 10 (maximum pain). The NRS demonstrates good reliability and responsiveness amongst a population of patients with AKP (Bennell et al. 2000; Crossley et al. 2004; Green et al. 2014).

#### Lower extremity functional scale questionnaire

The lower extremity functional scale (LEFS) consists of 20 items that measure the ability to perform various functional activities and activities of daily life. The LEFS is scored out of a maximum score of 80 and a higher score indicates a higher level of function. The LEFS demonstrates high reliability and responsiveness in the population of patients with AKP (Crossley et al. 2004; Watson et al. 2005).

#### Criteria for positive and negative knee joint position sense

The main outcome measurement for knee JPS testing was AE. Absolute error refers to the difference between the test or TA and the reproduced angle. Absolute error represents accuracy without directional bias. For our study, abnormal JPS was defined as an AE equal or greater than five degrees. This criterion was based on studies by Relph and Herrington (2015, 2016) and Clark et al. (2016) using healthy pain-free participants. The mean AE from five trials for each test was used for statistical analysis (Selfe et al. 2006). Relative error (RE) outcomes are also presented. Relative error is the difference between the TA and the response angle with directional bias. Directional bias refers to the participant being able to reach the TA or undershooting, which could result in a negative RE (Lokhande et al. 2013).

### Testing procedures

#### Initial screening

**FIGURE 1:**
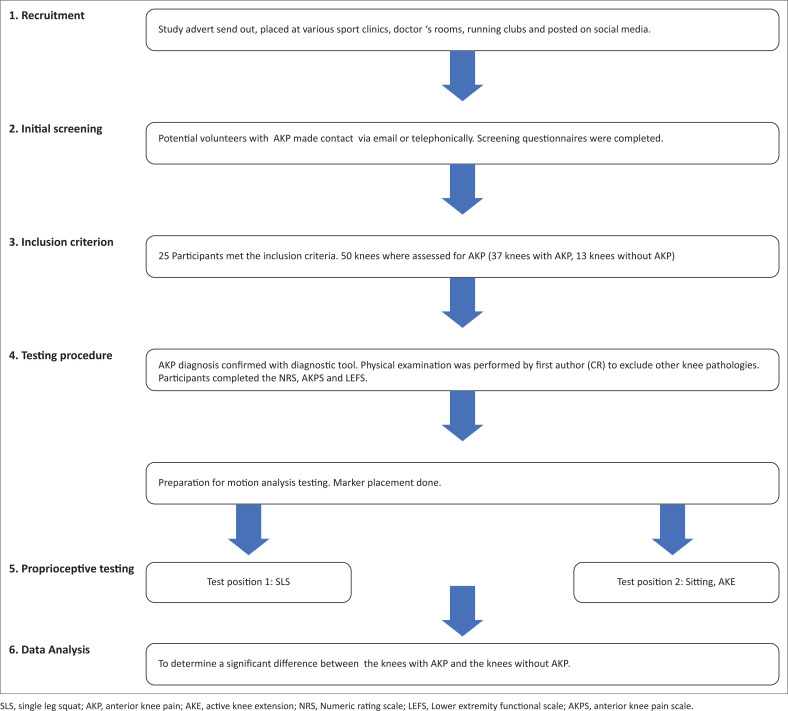
Flow diagram of study procedure.

To verify that potential participants met the inclusion criteria they completed a screening questionnaire via email. If they met the criteria they were then booked for a physical examination and movement analysis assessment session. Prior to JPS testing, participants completed the AKPS questionnaire and the LEFS questionnaire. A data collection form was used to collect participants’ personal details and variables including age, gender, body length, episodes and duration of AKP, area of symptoms, type of treatment received for AKP and sports participation. A flowchart of the study procedure can be seen in Figure 1.

#### Physical examination and diagnosis

The diagnostic checklist was completed, and the physical examination was performed to confirm a diagnosis of AKP and to exclude other knee pathologies prior to testing. The physical examination (P/E) was conducted by the first author (CR). Additionally, anthropometrics (weight, BMI, leg length) were measured using a digital scale, a measuring tape and calipers for each participant.

#### Preparation for Vicon testing

Participants were dressed in shorts and were barefoot and were requested to shave their legs the night prior to the assessment and to not have any lotion on their skin on the assessment day to ensure effective marker placement. Thirty retro-reflective markers were placed on bony landmarks according to the lower limb plug-in gait model (Clark et al. 2016). Additional pelvic markers, a sacral wand, two extra shin markers and extra anterior and posterior thigh markers were added, to ensure JPS accuracy. The first author (CR) performed the marker placement assisted by a research assistant. Reflective markers were placed in standing in preparation for SLS and re-applied with the participant seated to ensure accurate positioning of markers placements for AKE. Static and dynamic calibrations were performed in both test positions.

#### Pain measurement

During the test trials participants were asked to verbally indicate the severity of their AKP using the NRS pain scale. Pain severity was measured at the start and end of proprioceptive testing.

#### Proprioceptive testing

All participants were familiarised with the proprioceptive test procedure by means of explanation, demonstration and a practice opportunity. The participants were asked to resume the test position that is, (1) standing or (2) sitting. The TA was determined by each participant according to his or her capabilities and comfort level, that is, the TA was unique to each participant.

### Single leg squat

#### Starting position

**FIGURE 2:**
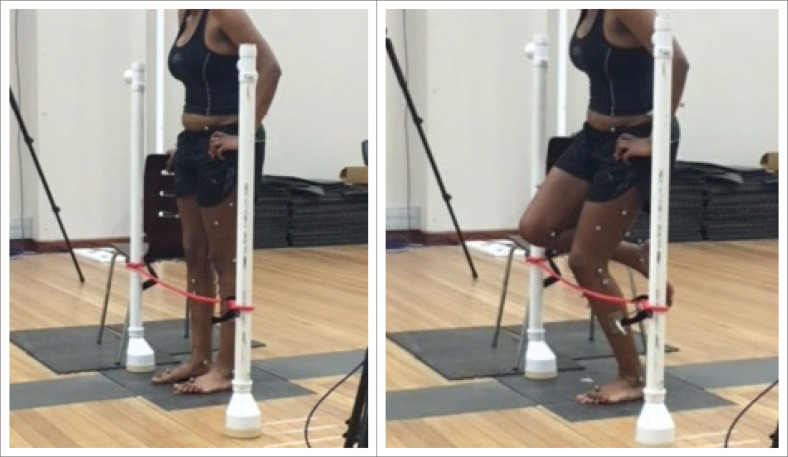
Single leg squat in standing to measure weightbearing joint position sense.

For the SLS, participants supported one hand on a chair for balance. They stood on the tested leg, whilst the other leg was slightly flexed at the hip and knee in a position that was comfortable (Figure 2).

#### Instructions to participant

Participants were asked to do a SLS and stop in the mid-range and to briefly hold this mid-range angle to position the H-frame indicating this angle as the TA.

#### Test trial

Participants were cued to squat down till they felt the cross bar of the H-frame and instructed to hold the SLS for 5 s to establish and familiarise themselves with the TA. The test trial was repeated five times.

#### Test procedure

Participants were then blindfolded, and the H-frame was removed. They were instructed to perform a SLS, and to indicate when they had reached the TA by shouting ‘stop’. This position indicated the reproduced angle and was maintained for 5 s to record the data. Testing was repeated five times. This number of repetitions has been recommended for JPS testing (Selfe et al. 2006). Single leg squat was repeated on the knees without AKP for comparison.

### Sitting: Active knee extension

#### Starting position

**FIGURE 3:**
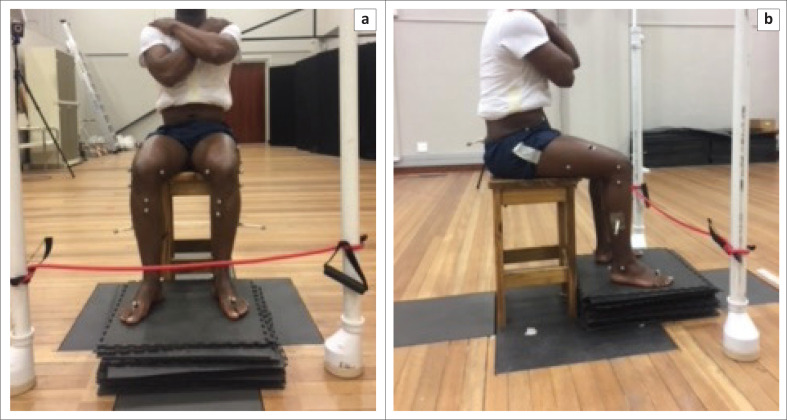
Active knee extension in sitting (a) frontal view and (b) lateral view to measure non-weightbearing joint position sense.

Participants were seated on an 800 mm high bar stool, with both feet supported and were positioned with the popliteal fossa approximately 5 cm from the edge of the chair. Their arms were crossed over their chests comfortably to avoid obstruction of the pelvic markers (see Figure 3).

#### Instructions to participants

Participants actively extended the knee through the range of 90° knee flexion to 0° knee extension; thereafter they had to stop in the mid-range position. They were asked to briefly hold this mid-range angle to position the H-frame indicating this position as the TA. They were verbally cued to resume the starting position.

#### Test trial

Participants actively extended the knee from the starting position of 90° knee flexion to the TA and were verbally cued to hold this position for 5 s to establish the TA. Thereafter, they were asked to return to the starting position of the knee in 90° flexion. The test trial was repeated five times.

#### Testing

The H-frame was removed at the commencement of AKE testing. Participants were asked to repeat AKE, indicating when the TA had been reached, by shouting ‘stop’. This position indicated the reproduced angle and participants maintained this position for 5 s. Testing was repeated five times. The AKE was repeated on the knees without AKP for comparison.

### Data analysis

All descriptive data (demographic information, functional and pain scales) were analysed using statistics to indicate central tendencies (means and standard deviations). For JPS data, a non-parametric test approach was adopted as these data were not normally distributed. Data were captured through the Vicon Nexus 3D motion analyses system. Descriptive JPS data were analysed using statistics to indicate central tendencies in data (medians and interquartile ranges). Joint position sense scores were also categorised as ‘impaired’ if the AE was greater than five degrees or ‘not impaired’ if the AE was less than five degrees. Chi-square calculations were then performed for the categorical data to determine differences in JPS between the participants’ knees with AKP and the participants’ knees without AKP during SLS and AKE depending on the proportions of participants’ knees in the two categories of impairment. The alpha level was set at *p* < 0.05.

### Ethical consideration

Ethical approval was obtained from the Health Research Ethics Committee of Stellenbosch University under reference number S16/10/197. This article is based on and adapted from a master’s degree thesis by Carlyn Rhode (2018) entitled, ‘Proprioceptive differences in individuals with anterior knee pain’. The thesis is available at https://scholar.sun.ac.za/handle/10019.1/103620 and the author has consented to the submission of this article. The study protocol was registered on the Clinicaltrials.gov database under ID number NCT03998241. This record is available online at https://clinicaltrials.gov/ct2/show/study/NCT03998241.

## Results

### Sample description

A total of 25 participants complied with the inclusion criteria (Table 1); with the majority being female (*n* = 22).

**TABLE 1:** Sample description for included participants (*n* = 25).

Variables	*n*	%
**Gender**		
Female	22	88
Male	3	12
**Age**		
Mean (SD)	27.8	7.6
Range (min – max)	14–40	-
**BMI (kg/m^2^)**		
Mean (SD)	28.2	7.4
Range (min – max)	20.9–45.7	-
**Activity levels of participants**		
Sedentary	6	24
Physically active	19	76
**Usual knee pain (NRS)**		
Mean (SD)	4.5	2.0
Range (min – max)	0–9	-

SD, standard deviation; BMI, body mass index; NRS, numeric rating scale.

The participants had a mean age of 27.8 years and a mean BMI of 28.2 kg/m^2^ (range of 20.9 kg/m – 45.7 kg/m).

A total of 12 (52%) participants reported having AKP symptoms in both knees and 10 (40%) reported their right knee as most affected. A total of 19 (76%) reported being physically active and 6 (24%) were sedentary. The participants’ mean usual pain level according to the NRS was 4.5/10.

The participants’ duration of symptoms ranged from 2 months to 11 years with a mean duration of 28.4 months (Table 2). The areas of symptoms were predominantly in the front of the patella (*n* = 10; 40%) or in front and just below the patella (*n* = 10; 40%). Five (20%) participants reported their area of symptoms to be behind the patella. The most frequently reported aggravating activities were squatting (*n* = 19; 76%), prolonged sitting (*n* = 10; 40%) and climbing stairs (*n* = 11; 44%).

**TABLE 2:** Participant symptom presentation and activity level (*n* = 25).

Variables	*n*	%
**Duration of symptoms (months)**		
Mean (SD)	28.4	32.6
Range (min – max)	2–132	-
**Area of symptoms**		
Front of patella	10	40
Front and below patella	10	40
Behind patella	5	20
**Aggravating activities**		
Squats	19	76
Prolonged sitting	11	44
Running	3	12
Kneeling	6	24
Lunging	4	16
Going up stairs	11	44
Going down stairs	2	8
Going up and down stairs	4	16
**Exercise per week**		
Mean (SD)	2.8	2.0
Range (min – max)	0–6	-
**Sport participation**		
Gym	12	48
Running	11	44
Dance	2	8
**Functional limitations**		
Can do all ADL	15	60
Stopped physical activity	9	36
Other (struggles to stay active)	1	4

ADL, Activities of daily living; SD, standard deviation.

### Pain and function

The participants’ AKPS ranged from 52 to 92 with an average of 72 out of 100 points. A score of 70–100 represents moderate disability (Singer & Singer 2009). The LEFS ranged from 31 to 77 with an average of 58 out of 80 points indicating moderate functional impairment. Participants reported that pain levels during the proprioceptive testing procedure ranged from 0 to 9/10 on the NRS scale with a mean pain level of 4.5 out of 10.

### Physical examination findings

The aggravating functional activities that reproduced the participants’ known AKP symptoms were predominantly squats (*n* = 25; 100%), going both up and down stairs (*n* = 5; 20%) and going down stairs, respectively (*n* = 4; 16%). During the physical examination of the PFJ, passive accessory movements easily reproduced their symptoms. A positive patellar compression test was reported by 22 (88%) patients. Other PFJ accessory movements were also positive in 11 (44%) and palpation of the patella border reproduced 6 (24%) of the participants’ pain.

### Joint position sense results

#### Single leg squat, comparing knees with anterior knee pain and knees without anterior knee pain (in participants with anterior knee pain)

The JPS results comparing participants’ knees with and without AKP during SLS are summarised in Table 3. The median TA of the knees with AKP (*n* = 37) was 38.5 degrees compared with 47.1 degrees in the knees without AKP (*n* = 13). Those with AKP demonstrated a larger variation in IQR compared with those without AKP. When comparing the AE the participants’ knees with AKP had a smaller AE compared with those knees without AKP. However, the participants’ knees without AKP demonstrated a greater variation in the IQR of the AE. The median AE of both groups was less than five degrees, indicating that proprioception was not impaired. The RE was larger in those knees with AKP (-3.8 degrees) compared with knees without AKP (-2.7) with a similar IQR (6.7) compared with the knees without AKP (7.3).

**TABLE 3:** Joint position sense results between the knees with anterior knee pain and without anterior knee pain (in participants with anterior knee pain).

Variables	SLS			AKE		
	Target angle (TA)	Absolute error (AE)	Relative error (RE)	Target angle (TA)	Absolute error (AE)	Relative error (RE)
**Knees with AKP (*n* = 37)**						
Median	38.5	3	−3.8	31.6	3.1	−3.2
IQR	10.1	2.7	6.7	14.7	2.6	6.3
**Knees without AKP (*n* = 13)**						
Median	47.1	3.9	−2.7	34.5	4.4	2.6
IQR	10.8	6.1	7.3	6.3	1.7	7.2

SLS, single leg squat; AKP, anterior knee pain; IQR, interquartile range; AKE, active knee extension.

#### Sitting: Anteriorctive knee extension, comparing knees with anterior knee pain to knees without anterior knee pain (in participants with anterior knee pain)

The JPS results comparing participants’ knees with and without AKP during AKE are summarised in Table 3. The median TA of the participants’ knees with AKP (*n* = 36) was 31.6 degrees compared with 34.5 degrees in the knees without AKP. The AE was slightly greater in the knees without AKP (AE = 4.4 degrees) compared with those with AKP. The median AE of both groups was less than five degrees, indicating that proprioception was not impaired. However, there was a difference when comparing the IQR of the AE between the two groups. The group with AKP displayed greater variability in IQR compared with the group without AKP for the TA and AE. The RE was greater in the group of knees without AKP (2.6 degrees) compared with the group of knees with AKP (–3.2 degrees). There was a similar variation in IQR of knees with AKP (6.7) compared with the knees without AKP (7.3).

A total of 37 knees (37/50) presented with AKP. During SLS 10 (27%) of the participants’ knees with AKP presented with altered JPS with an AE equal or greater than five degrees. Twenty-seven (73%) with AKP had an AE of less than five degrees. During AKE 10 (27%) participants with AKP presented with altered JPS with an AE equal or greater than five degrees. Thirteen of the 50 knees (26%) had no symptoms of AKP (knees without AKP). During SLS, 6 of the 13 (46%) without AKP had altered JPS with an AE equal or greater than five degrees. During AKE, 4 of the 13 (30%) without AKP presented with altered JPS with an AE equal or greater than five degrees. The remaining nine participants (69%) in this group had an AE less than five degrees, indicating that JPS was not impaired.

A chi-square test of independence was performed to examine the relationship between pain and impaired JPS (Table 4). The relationship between these variables during a SLS was not significant, *X*^2^ (1, *N* = 50) = 1.7, *p* = 0.2. The relationship between these variables during AKE was not significant, *X*^2^ (1, *N* = 50) = 1.07, *p* = 0.08. Therefore, the painful knees in participants with AKP were not more likely to present with altered JPS compared with the non-painful knee in participants with AKP during both activities (*p* > 0.05).

**TABLE 4:** Knees with anterior knee pain compared with knees without anterior knee pain during single leg squat and active knee extension.

Variables	Chi-square calculation during SLS				Chi-square calculation during AKE			
	Impaired (AE > 5 degrees)		Not impaired (AE < 5 degrees)		Impaired (AE > 5 degrees)		Not impaired (AE < 5 degrees)	
	*n*	%	*n*	%	*n*	%	*n*	%
**Knees with AKP (*n* = 37)**								
Knees (%)	10	27	27	73	10	10.29	27	73
**Knees without AKP (*n* = 13)**								
Knees (%)	6	46	7	54	4	31	9	69

**Totals (% out of 50)**	**16**	**32**	**34**	**68**	**14**	**28**	**36**	**72**

Note: Chi-square statistic for Chi-square calculation during SLS: X^2^ (1, *N* = 50) = 1.7, *p* = 0.2. Chi-square statistic for Chi-square calculation during AKE: X^2^ (1, *N* = 50) = 0.07, *p* = 0.8.

SLS, single leg squat; AKP, anterior knee pain; AKE, active knee extension.

## Discussion

Joint position sense in people with AKP remains a controversial topic. Our study which included a young cohort of South Africans showed that there were no significant differences in knee JPS when comparing the knees with AKP to those without AKP during SLS or the AKE positions and adds to the knowledge base of JPS in young people with AKP. However, a subgroup of participants with AKP and impaired JPS bilaterally was identified. Approximately a third of the study participants were in this subgroup. In addition, there was a tendency towards a larger knee flexion in the TA of knees without AKP. This could be because increased knee flexion has been linked to increased pain in individuals with AKP because of increased PFJ contact stress (Besier et al. 2005).

Research into AKP and JPS has yielded inconsistent findings. Some studies concur with our findings of no significant difference in the perception of JPS in knees with and without AKP (Bennell et al. 2005; Naseri & Pourkazemi 2012; Yosmaoglu et al. 2013). These studies also used JPS to evaluate knee proprioception and calculated the AE to express JPS. On the contrary, others have found that JPS is more affected in people with AKP compared with individuals without AKP (Akseki et al. 2008; Baker et al. 2002; Cyrillo & Cabral 2014; Guney et al. 2015). These contradictory findings could be a result of differences in study methodology and participant characteristics.

Some of the unaffected knees in participants with AKP also had altered JPS according to the classification criteria (Callaghan et al. 2002). Similar results have been reported when comparing JPS in individuals with AKP compared with healthy controls (Baker et al. 2002; Cyrillo & Cabral 2014). These authors found the perception of JPS to be altered in those with AKP and in healthy controls without AKP. The interpretation of altered JPS in unaffected knees of participants with unilateral AKP is unclear but could be because of compensatory mechanisms during gait in people with AKP (Barton et al. 2009). In an attempt to decrease PFJ reaction forces and to avoid pain, individuals with AKP may develop a quadriceps avoidance gait pattern (Sanchis-Alfonso et al. 2016).

We defined abnormal knee JPS as an AE equal or greater than five degrees. This criterion was based on a study by Callaghan et al. (2002), who tested the criterion on healthy participants. It must be considered that classification of abnormal JPS has not been used in a population affected with AKP, making the interpretation of the findings difficult in this population (Callaghan 2011). The group of knees without AKP was smaller than the knees with AKP making it very difficult to compare the proprioceptive findings. Inter-subject comparisons for each participant’s proprioceptive outcomes were not compared and should be considered in future studies.

The observation that a small percentage of the knees with AKP and the knees without AKP had an AE greater than five degrees cannot be generalised to the rest of the population because of the small sample group.

We included participants with unilateral and bilateral AKP, which differs from other studies that used control groups without AKP for comparison. Anterior knee pain symptoms in our study were not restricted to just one knee, as more than half the individuals reported both knees being symptomatic, as AKP can be present in both knees (Kurt et al. 2016). If the pathological and normal knees are affected this could indicate that JPS should be considered in the aetiology in this subgroup (Akseki et al. 2008). If this is the case, addressing JPS in the early phases of rehabilitation might be useful to prevent chronicity of symptoms.

Studies with similar findings have included an athletic population (Naseri & Pourkazemi 2012), female athletes diagnosed with AKP (Yosmaoglu et al. 2013) and healthy participants with induced AKP (Bennell et al. 2005). Compared with pain-free controls, AKP is unlikely to affect the knee JPS in athletes (Naseri & Pourkazemi 2012). This could depend on the severity of knee pathology, pain intensity and level of physical activity amongst the athletes. Furthermore, it is unclear whether their proprioceptive abilities were significantly affected or not by the severity of knee pathology and pain intensity and whether or not their proprioceptive (JPS) ability was improved by functional state. Athletes with moderate pain may not present with proprioceptive deficits, as higher levels of motor function could account for increased proprioceptive feedback from adjacent joints and muscles (Naseri & Pourkazemi 2012; Smith et al. 2013). Therefore, it is possible that only more sedentary participants with AKP present with altered JPS.

Our participants were included irrespective of their participation in sport or activity levels, whereas other studies include athletes or restrict the population to only women diagnosed with AKP. The literature highlights that AKP is common amongst active individuals and has a higher incidence in female athletes (Neal et al. 2016; Prins & Van Der Wurff 2009). Our population is reflective of these findings, with females constituting the majority of our study population: two-thirds were active individuals and 44% where runners.

The severity of the AKP pathology, participants’ pain levels and activity levels are thought to have influenced proprioceptive function in these studies. The pain levels of our participants ranged between zero to nine out of 10 with a mean pain level of 4.5/10. It can be argued that in order to influence proprioceptive abilities, pain severity levels need to be higher than moderate (Naseri & Pourkazemi 2012).

Joint position sense was tested in both a WB (SLS) and NWB position (AKE). Other studies predominantly tested JPS in only a NWB position (Akseki et al. 2008; Bennell et al. 2005; Cyrillo & Cabral 2014).

Weightbearing test positions are more likely to be relevant in an AKP population as this is when patients typically experience pain. However, JPS in WB positions need to be measured accurately and reliably. In more recent studies, the Biodex 360 has been used to measure knee JPS. The Biodex 360 is considered a reliable measurement tool to evaluate knee JPS, however it can only account for a NWB test position.

Vicon 3D motion analyses allowed for testing knee JPS during both standing and sitting. The Vicon 3D motion analysis system is regarded as the gold standard in motion analyses (Ehara et al. 1997; Richards 1999). This is the first study to make use of the Vicon 3D motion analyses to assess knee JPS in an AKP population, which allows for better measurement accuracy and a WB assessment of JPS. Joint position sense testing in a WB position has been found to be more reliable and accurate for JPS compared with NWB test procedures (Stillman & McKeeken 2001). However, NWB testing had a greater potential to isolate the proprioceptive status of the knee joint only. Therefore, it is recommended to use a combination of both positions.

### Limitations

There is still limited research on investigating JPS changes in individuals with AKP and conflicting results remain a cause for concern. We estimated the sample size using previous studies and this resulted in a small sample size. Future studies should use a more accurate approach such as using the minimal detectable difference to estimate the sample size. The use of larger sample groups and being able to match cases to healthy controls with unaffected knees should also be addressed in future studies. A larger sample size would also enable analysis of JPS in different subgroups of participants with AKP and allow for more consideration to be given to the influence of age, gender and duration of symptoms. There may be a subgroup of patients who have both AKP and poor JPS (Callaghan 2011), which is cause and which is effect, remains uncertain, until prospective studies are undertaken.

Only JPS testing was performed, which is only one aspect of proprioceptive testing. The best test procedure still needs to be described to assess JPS, as it remains challenging to draw conclusions because of variations in testing methods and measurement tools. Although participants with a high BMI were not excluded in our study, future studies should consider excluding obese participants with a BMI greater than 30, for a couple of reasons.

Excess soft tissue influences the reliability of the motion analysis as it can cause movement of the markers and wands during motion capture procedures, and the extra weight itself could lead to the development and chronicity of the knee symptoms because of the increased loading of the knee joint.

Frontal plane mechanics were not considered, and it is, therefore, unclear how abnormal biomechanics correlate with JPS findings. This should be addressed in future studies. Nakagawa, Maciel and Serrão (2015) proposed that an increased dynamic Q-angle during SLS may indicate an inability of the individual to stabilise the lower limb in the frontal plane.

## Conclusion

We investigated JPS in individuals with AKP and found that JPS is not significantly more impaired in knees with AKP compared with knees without AKP. The use of the Vicon 3D motion analysis system added to the measurement precision. Our findings suggest that a subgroup of individuals with altered JPS may exist in an AKP population, however, longitudinal studies are needed to establish how altered JPS relates to the pain experienced.
